# The association between adherence to diet quality index and cardiometabolic risk factors in overweight and obese women: a cross-sectional study

**DOI:** 10.3389/fpubh.2023.1169398

**Published:** 2023-07-13

**Authors:** Azam Mohamadi, Farideh Shiraseb, Atieh Mirzababaei, Assa AkbarySedigh, Moloud Ghorbani, Cain C. T. Clark, Yasaman Aali, Khadijeh Mirzaei

**Affiliations:** ^1^Department of Community Nutrition, School of Nutritional Sciences and Dietetics, Tehran University of Medical Sciences (TUMS), Tehran, Iran; ^2^Department of Clinical Nutrition, School of Nutritional Science Dietetics, Tehran University of Medical Sciences, Tehran, Iran; ^3^Department of Community Nutrition, Faculty of Nutrition and Food Sciences, Tabriz University of Medical Sciences, Tabriz, Iran; ^4^Centre for Intelligent Healthcare, Coventry University, Coventry, United Kingdom

**Keywords:** ABSI, cardiometabolic risk factors, DQI, dietary intake, obesity

## Abstract

**Background:**

Obesity and overweight status increase the risk of cardiovascular disease. Diet quality can also predict the risk of cardiovascular diseases in obese and overweight patients. Therefore, in this study, we sought to examine the relationship between diet quality index (DQI) and cardiometabolic risk factors in obese and overweight women.

**Method:**

A cross-sectional study was conducted on 197 Iranian women with a Body Mass Index (BMI) > 25, 18–48 years, and recruited from 20 Tehran Health Centers. Nutrition intake and DQI were assessed using a 147-item semi-quantitative food frequency questionnaire (FFQ). Additionally, anthropometric measurements, body composition, biochemical evaluations, and cardiometabolic risk factors were evaluated.

**Results:**

There was an association between DQI and waist-to-hip ratio (WHR), atherogenic index of plasma (AIP), and CHOLINDEX in obese women, after adjusting for potential confounders. Whereas, there were no significant associations of the tertiles of DQI compared with the first tertile in other cardiometabolic risk factors, before and after adjustment.

**Conclusion:**

This study provides evidence that dietary intake and DQI are associated with cardiometabolic risk factors and that dietary modification may be a predictor for reducing WHR, AIP, and CHOLINDEX. However, more research is needed to develop a DQI that reflects changes in cardiometabolic risk factors by considering women's eating habits and patterns.

## Introduction

Cardiovascular disease (CVD) has been reported as being responsible for 46% of all deaths and 20–23% of the burden of disease in Iran in 2019 (1). The term cardiometabolic risk refers to clinical abnormalities, including obesity, diabetes, and high blood pressure, that should be diagnosed and treated early (2). Furthermore, young women have increasingly prevalent risk factors for CVD (3). However, it is evident that women are less likely to receive preventive treatment, relative to men, and is influenced by behaviors, environment, lifestyle, and nutrition (3).

There are several important cardiometabolic risk factors, including lipid accumulation product (LAP), which is associated with glucose intolerance, CVD, and MetS (4), while the hypertriglyceridemic waist (HW) phenotype (5) is a clinical indicator of visceral obesity diagnosis that may be related to coronary risk factors among women (6). AIP and atherogenic coefficient (AC) are early biomarkers of CVD in developing countries (7), and Castelli Risk Index 1 (CRI-I) and CRI-II are additional, independent, and more precise, risk factors in comparison with traditional lipid parameters (8). Furthermore, an additional simple index, namely CHOLINDEX, considering three main cholesterol parameters, low-density lipoprotein (LDL), high-density lipoprotein (HDL), and triglycerides (TG) levels, has been developed to evaluate cardiometabolic risk (9).

Another relevant predictor of health status is ABSI, an alternative anthropometric measurement that considers height, weight, and waist circumference (WC) and thus more representative of abdominal adipose tissue, and a significant risk factor in predicting premature mortality (10). In line with this, cohort studies have indicated that diabetes and mortality could be predicted by ABSI (11, 12). However, Maessen et al. showed that ABSI was not significantly associated with CVD risks, as compared to BMI and WC in adults (13), and among Iranian adults, it was concluded that ABSI was a weak predictor for CVD risks and MetS (14). In a cohort study conducted on 8,248 participants (4,471 women), the authors aimed to examine whether ABSI could improve the predictive performances for CVD of Framingham's general CVD algorithm. The results showed that ABSI was associated with an increased risk of incident CVD among both men and women but could not improve the predictability of the Framingham algorithm (15). In addition, the triglyceride glucose index (TyG) is one of the methods by which insulin resistance can be estimated (16). Furthermore, it has been indicated that TyG may be associated with high blood pressure, vascular stiffness, and coronary artery calcification (17–19).

Modifiable factors, including physical inactivity and nutrition, may contribute to accelerating cardiometabolic risk (20, 21). Earlier research has largely been concerned with the role of single nutrients and foods in chronic diseases; however, contemporary research has tended to assess the total diet (22, 23). To have a better understanding of overall eating patterns and behaviors in a population, the diet quality index (DQI) has been developed and evaluated (24). In postmenopausal women, it has been demonstrated that a low-quality diet, including the low intake of vegetables and fruits and excessive consumption of sodium, has detrimental impacts on cardiometabolic risk factors, such as abdominal obesity (25). A cross-sectional study based on the Australian Diabetes, Obesity, and Lifestyle study indicated that higher diet quality was significantly associated with cardiometabolic risk factors, including lower blood pressure, lower fasting plasma glucose, and greater insulin sensitivity among men and women (26). Moreover, it was reported that a higher DQI score at baseline was inversely associated with WC, TG, TG to HDL ratio, and the total cholesterol to HDL ratio after 9 years in Spanish adults (27). To our knowledge, the association of DQI with cardiometabolic risk factors and ABSI has scarcely been investigated. Therefore, in this study, we sought to examine the relationship between diet quality index (DQI) and cardiometabolic risk factors in obese and overweight women.

## Subjects and methods

This cross-sectional study was conducted using 197 women recruited from 20 Tehran Health Centers, using simple random sampling. The inclusion criteria were as follows: aged from 18 to 48 years, BMI ≥25 kg/m^2^, without a history of hypertension, no intake of alcohol and opiate drugs, not being pregnant, and not having an acute or chronic infection; and the exclusion criteria were having a history of CVDs, thyroid, cancer, diabetes, liver disease, kidney disease, and smoking. In addition, participants who had been following any arbitrary special dietary regimen, those with chronic disease(s) affecting their diet, or if their daily energy intake was < 800 or >4,200 kcal (28) were excluded. All participants were asked to sign the written consent before the study commencement, and the study was approved by the ethics committee of Tehran University of Medical Sciences (R.TUMS.VCR.REC.1395.1234).

### Anthropometric measurements

The body compositions, including weight, fat and lean mass, and WHR, of the female participants were assessed by the bioelectric impedance analyzer (In Body 770 scanner, Korea) (29). In addition, height was measured to the nearest 0.5 cm to evaluate BMI as weight (kg) divided by height (m^2^). WC measurement was performed at the point of the umbilicus after exhalation. According to the World Health Organization (WHO) criteria for the classification of weight, a BMI of ≥ 25 kg/m^2^ was considered overweight, and a BMI of ≥ 30 kg/m^2^ was considered obesity (30).

### Biochemistry measurements

Blood samples were drawn after 12 h of overnight fasting to assess LDL, HDL-C, TG, fasting blood sugar (FBS), and total cholesterol by Pars Azmoon laboratory kits (test Pars Inc, Tehran, Iran). The serum was separated and stored at a temperature of −70°C until the analyses were carried out after centrifugation. Levels of TG, total cholesterol, HDL-C, LDL-C, and fasting blood sugar (FBS) were measured using glycerol-3-phosphate oxidase phenol 4-amino antipyrine peroxidase (GPO-PAP), enzymatic endpoint, direct enzymatic clearance, and glucose oxidase phenol 4-amino, respectively.

### Cardiometabolic risk factors calculation

ABSI = $\frac{\text{WC}}{\;{\text{BMI}}^{2/3}\;{\text{height}}^{1/2}\;}$ (10);
LAP index =(WC (cm)-58) × TG (mmol) (31);
AIP: $\log_{10}^{\text{TG}/\text{HDL}-\text{C}}$ (32);
AC: (TC– HDL)/HDL (8);
CRI-I $=\frac{\text{Total}\;\text{Cholesterol}}{\text{HDLc}}$, respectively (33);
CRI-II $=\;\frac{\text{LDLc}}{\text{HDLc}}$, respectively (33);
TyG = Ln [fasting triglycerides (mg/dL) × fasting glucose (mg/dL)/2] (16);
CHOLINDEX =LDL-C-HDL-C (TG < 400 mg/dL), LDL-C-HDL-C + 1/5 of TG (TG ≥ 400 mg/dL) (9);
TyG-BMI (triglyceride glucose index- body mass index) = Ln [fasting plasma glucose (mg/dL) × fasting triglycerides (mg/dL)/2] × BMI (34);
TyG-WC (triglyceride glucose index- waist circumference) = Ln [fasting plasma glucose (mg/dL) × fasting triglycerides (mg/dL)/2] × WC (35).
The HW phenotype was defined by the presence of increased WC (>88 percentile by age and sex of the sample itself) and increased serum triglycerides (>100 mg/DL) (36).

### Dietary intake measurement and DQI calculation

The dietary intake of the women was collected by an instructing nutritionist via a face-to-face interview with a 147-item semi-quantitative food frequency questionnaire (FFQ), where its validity and reliability were previously avowed in the Iranian population (37). The average consumption frequency was considered over the past year on a daily, weekly, and monthly basis. Household measures were taken into account for portion sizes and then converted to grams (38). The food composition table (FCT) of the United States Department of Agriculture (USDA) was used to evaluate energy and nutrients. The Iranian FCT was considered for local foods that were not present in the USDA FCT. There are seven food groups considered in the DQI, which are as follows: fast food (four items); vegetables and fruit (seven items); legumes, chicken, soy protein or fish (four items); sweets (six items); butter, hydrogenated oil, animal fats, or ghee (four items); egg, whole dairy products, or meat (four items); and olive and non-hydrogenated oil (two items). The calculation of this index is based on adequacy, variety, moderation, and balance (39). Variety was assessed by two components: “between-food groups” (0–15 points) and “within-protein source group” (0–5 points). Adequacy evaluates fruits, vegetables, grains, protein, fiber, calcium, iron, and vitamin C intakes (40 points). Moderation assesses total fat, saturated fat, cholesterol, sodium, and empty-calorie foods (30 points). The balance of the micronutrient distribution in the diet and the fatty acid ratio are also examined (10 points). Details about the scoring method of DQI were published previously (40). The whole DQI score is 100, such that a higher score indicates a better quality of the diet. Ultimately, the DQI score of participants was categorized based on tertiles.

### Physical activity (PA) measurement

A valid and reliable international PA questionnaire (IPAQ), designed by the WHO and validated in Iranian women adults, was used to assess PA levels (41). The participants were asked to answer questions such as the time they spent on walking, moderate, and vigorous PA during the last week. Then, the time of each PA was converted to minutes per week and calculated as the metabolic equivalent of the task (MET/minutes/week).

### Other covariates assessments

Demographic characteristics, including age, marital status, supplement consumption (multivitamins and minerals), socioeconomic status, education, and occupation status were collected.

### Blood pressure measurements

Systolic and diastolic blood pressure (SBP and DBP) were evaluated after 15-min rest, using a mercury sphygmomanometer.

### Statistical analysis

In this study, SPSS software version 26 (Chicago, USA) was used for all data analysis, and a *p*-value of < 0.05 was considered statistically significant. Participants were categorized according to tertiles of DQI. Kolmograph–Smirnov test and histogram inspection were used to determine the normal distribution of the data. All variables with normal distribution were analyzed by parametric tests, where one-way analysis of variance (ANOVA) for continuous variables and chi-square analysis for categorical variables were used to compare subject characteristics and dietary intake across tertiles of DQI score, reported as mean (SD). Analysis of covariance (ANCOVA) was used to examine demographic characteristics, anthropometric measurements, clinical assessments, and dietary intake across tertiles of DQI score via adjusting for age, BMI, physical activity, and energy intake in model 1 and education, economic status, and supplement consumption in model 2. Notably, BMI, as a colinear variable, was not adjusted for outcomes including BMI, BFM, WHR, WC, TyG-BMI, ti, LAP, and ABSI in model 1. To examine the association between DQI score and cardiometabolic risk factors, a binary logistic regression model was applied and reported as β and 95% confidence interval (CI).

## Results

### Study population characteristics

In total, 197 women with a mean age and BMI of 35.5 years and 30.5 kg/m^2^, respectively, participated in this study. The baseline characteristics of participants were presented in Table 1.

**Table 1:** Baseline characteristics of participants.

**Variables**	**Mean**	**SD**
ABSI	0.078	0.002
AIP	0.348	0.235
TyG	8.376	0.498
CHOLINDEX	50.240	22.166

ABSI, a body shape index; AIP, Atherogenic index of plasma; TyG, triglyceride glucose index.

All data are presented as mean and SD.

### Baseline characteristics of participants across the tertiles of DQI scores

The characteristics of participants across the tertiles of DQI scores in overweight and obese women are shown in Table 2. Participants with a higher score of DQI were more physically active (*P*: 0.002), before and after adjusting for age, energy intake, BMI, and physical activity. Interestingly, there was a statistically significant age difference among women across the tertiles of DQI scores after controlling for confounders (*P*: 0.011), but not in the crude model (*P*: 0.118). However, there were no significant differences between tertiles in other characteristics.

**Table 2:** General characteristics of participants across tertiles of DQI among overweight and obese women (*n* = 197).

	**T1 (*n* = 66)**	**T2 (*n* = 65)**	**T3 (*n* = 66)**	***P*-value**	*P*-value^*^
Age (year)	35.2 ± 9.5	33.9 ± 7.6	37.8 ± 7.4	0.118	**0.011**
Physical activity (MET/h/w)	733.2 ± 620.4	808.2 ± 676.4	1,551.6 ± 1,618.7	**0.002**	**< 0.001**
**Anthropometric measurements**					
Weight (kg)	77.5 ± 10.7	80.0 ± 11.5	78.8 ± 11.1	0.587	0.503
Height (cm)	159.4 ± 4.9	161.8 ± 6.0	160.2 ± 6.3	0.166	0.245
WC (cm)	110.8 ± 7.2	111.4 ± 7.0	112.5 ± 7.8	0.707	0.650
**Socioeconomic status**				0.556	0.692
Poor	14 (21.2)	11 (16.9)	13 (19.6)		
Moderate	21 (31.8)	32 (49.2)	28 (42.4)		
Good	31 (46.9)	22 (33.8)	25 (37.8)		
**Occupation status**				0.415	0.560
Unemployed	3 (4.5)	0 (0.0)	0 (0.0)		
Employed	63 (95.4)	65 (100.0)	66 (100.0)		
**Marital status**				0.583	0.176
Single	17 (25.7)	11 (16.9)	14 (21.2)		
Married	49 (74.2)	54 (83.0)	52 (78.7)		
**Education status**				0.793	0.710
Under diploma	6 (9.0)	5 (7.6)	3 (4.5)		
Diploma	19 (28.7)	22 (33.8)	18 (27.2)		
Bachelor and higher	41 (62.1)	38 (58.4)	45 (68.1)		
**Supplement use** ^£^				0.870	0.670
Yes	45 (68.1)	47 (72.3)	43 (65.1)		
No	21 (31.8)	19 (29.2)	23 (34.8)		

DQI, diet quality index; WC, waist circumference.

Values are mean ± standard deviation (SD) for continuous variables and number and percentage for dichotomous variables. Using one-way ANOVA for continuous variables and Chi-square test for categorical variables.

^*^P-value: adjusted for age, energy intake, BMI, and physical activity.

The categorical variables were reported in n (%).

^£^Multivitamins and minerals supplements were considered.

Bold values indicate *P*-value < 0.05 significant.

### Dietary intake of participants across the tertiles of DQI scores

The dietary intake of participants across the tertiles of DQI scores in overweight and obese women is presented in Table 3. Before adjusting for confounding factors, the consumptions of energy (*P*: 0.041), protein (*P*: 0.028), and carbohydrate (*P* < 0.001) increased from the first to the third tertiles of DQI. Women who adhered highly to DQI had a lower intake of total fat (*P*: 0.006), saturated fatty acids (*P*: 0.006), and mono-unsaturated fatty acids (*P*: 0.003); while, in contrast, results indicated a higher intake of iron (*P* < 0.001), calcium (*P*: 0.006), magnesium (*P* < 0.001), zinc (*P*: 0.006), selenium (*P*: 0.001), β carotene (*P*: 0.048), vitamin D (*P*: 0.049), vitamin B_1_ (*P*: 0.001), vitamin B_6_ (*P*: 0.009), folate (*P* < 0.001), and total fiber (*P* < 0.001). Regarding food groups, it should be mentioned that refined (*P*: 0.015) and whole (*P*: 0.002) grains, fruits (*P* < 0.001), vegetables (*P*: 0.001), and legumes (*P*: 0.001) were also consumed in higher quantities by women with a higher score of DQI. After controlling for energy intake, some nutrients remained statistically significant as follows: carbohydrate (*P* < 0.001), total fat (*P* < 0.001), saturated fatty acids (*P* < 0.001), mono-unsaturated fatty acids (*P* < 0.001), iron (*P* < 0.001), calcium (*P*: 0.039), magnesium (*P* < 0.001), zinc (*P*: 0.025), selenium (*P*: 0.029), thiamin (*P*: 0.001), folate (*P* < 0.001), and total fiber (*P* < 0.001). Also, whole grains (*P*: 0.009), fruits (*P*: 0.009), vegetables (*P*: 0.011), and legumes (*P*: 0.002) were the same as before adjustments. However, other nutrients showed no significant results across tertiles of DQI, before or after adjustment.

**Table 3:** Dietary intake of participants across tertiles of DQI among overweight and obese women (*n* = 197).

	**Mean** ±**SD**			***P*-value**	*P*-value^*^
	**T1 (*n* = 66)**	**T2 (*n* = 65)**	**T3 (*n* = 66)**		
Energy intake (kcal/d)	2,406.0 ± 753.3	2,785.5 ± 731.8	2,715.5 ± 658.3	**0.041**	–
**Macronutrients**					
Protein (g/d)	80.6 ± 34.8	92.8 ± 27.2	97.8 ± 22.6	**0.028**	0.189
Carbohydrate (g/d)	309.9 ± 93.6	393.0 ± 107.6	422.4 ± 122.0	**< 0.001**	**< 0.001**
Total fat (g/d)	100.0 ± 35.6	103.1 ± 35.4	80.8 ± 20.4	**0.006**	**< 0.001**
**Micronutrients**					
Cholesterol (mg/d)	258.3 ± 144.8	261.0 ± 104.9	220.9 ± 64.9	0.233	**0.006**
SFA (g/d)	31.5 ± 15.1	28.0 ± 9.7	23.1 ± 5.6	**0.006**	**< 0.001**
MUFA (g/d)	35.0 ± 13.3	33.2 ± 11.5	26.4 ± 7.0	**0.003**	**< 0.001**
Linoleic acid (g/d)	18.5 ± 8.6	19.0 ± 8.9	15.1 ± 5.4	0.079	**0.007**
Linolenic acid (g/d)	1.2 ± 0.5	1.2 ± 0.6	1.1 ± 0.6	0.684	0.170
Fe (mg/d)	15.6 ± 4.9	20.0 ± 5.7	21.5 ± 5.8	**< 0.001**	**< 0.001**
Calcium (mg/d)	1,011.7 ± 399.8	1,192.1 ± 398.2	1,295.8 ± 365.4	**0.006**	**0.039**
Mg (mg/d)	386.5 ± 134.4	479.5 ± 126.0	531.7 ± 141.6	**< 0.001**	**< 0.001**
Zn (mg/d)	11.4 ± 4.3	13.6 ± 4.2	14.4 ± 4.0	**0.006**	**0.025**
Selenium (mg/d)	102.2 ± 31.4	129.7 ± 42.2	135.0 ± 53.9	**0.001**	**0.029**
Vitamin A (RAE/d)	713.0 ± 460.7	837.2 ± 365.9	838.6 ± 343.7	0.262	0.889
β carotene	4,591.3 ± 3,540.6	5,416.5 ± 2,478.6	6,361.9 ± 3,234.0	**0.048**	0.195
Vitamin D (μg)	1.5 ± 1.2	2.3 ± 1.6	2.2 ± 1.7	**0.049**	0.226
Vitamin E (mg)	18.2 ± 107	19.1 ± 9.6	15.6 ± 6.0	0.248	0.126
Thiamin (mg)	1.8 ± 0.5	2.1 ± 0.5	2.3 ± 0.7	**0.001**	**< 0.001**
Vitamin B_6_ (mg)	1.9 ± 0.7	2.2 ± 0.6	2.3 ± 0.5	**0.009**	0.074
Folate (μg/d)	522.3 ± 147.7	614.4 ± 168.4	713.2 ± 183.7	**< 0.001**	**< 0.001**
Vitamin B_12_ (μg/d)	4.2 ± 1.9	4.8 ± 2.9	4.1 ± 1.5	0.294	0.302
Total fiber (g/d)	34.7 ± 15.0	46.5 ± 15.9	55.1 ± 17.4	**< 0.001**	**< 0.001**
**Food groups**					
Refined grain (g/d)	352.8 ± 148.9	456.9 ± 234.7	494.0 ± 276.3	**0.015**	0.068
Whole grain (g/d)	4.2 ± 4.8	6.1 ± 9.1	12.4 ± 15.1	**0.002**	**0.009**
Fruits (g/d)	359.5 ± 215.2	585.7 ± 312.9	590.1 ± 337.9	**< 0.001**	**0.009**
Vegetables (g/d)	343.2 ± 230.3	486.7 ± 234.2	549.8 ± 267.2	**0.001**	**0.011**
Nuts (g/d)	13.7 ± 17.6	15.4 ± 17.1	17.3 ± 18.7	0.677	0.621
Dairy (g/d)	319.0 ± 205.7	404.3 ± 225.5	432.3 ± 227.6	0.059	0.213
Legumes (g/d)	46.1 ± 27.1	45.6 ± 35.3	77.4 ± 56.6	**0.001**	**0.002**

MUFA, monounsaturated fatty acid; SFA, saturated fatty acid.

Values are mean ± standard deviation (SD) for continuous variables. Using one-way ANOVA for continuous variables and Chi-square test for categorical variables.

^*^Adjusted for energy intake.

Bold values indicate *P*-value < 0.05 significant.

### Association of DQI with cardiometabolic risk factors and ABSI

The associations of DQI with cardiometabolic risk factors in overweight and obese women are shown in Table 4. There were significant associations between DQI and WHR (β: −0.014, 95% CI: −0.039, 0.011, *P*-trend: 0.047), AIP (β: −0.086, 95% CI: −0.207, −0.006, *P*-trend: 0.027), and CHOLINDEX (β: −4.998, 95% CI: −15.597, 0.000, *P*-trend: 0.031) with the third tertile of DQI scores, as compared to people in the first tertile, in obese women after adjusting for age, BMI, PA, energy intake, education, socioeconomic status, and supplement use. However, in other cardiometabolic risk factors, no significant relationship was seen after adjusting for potential confounders. In the crude model, women who were in the second tertile of DQI had a lower score of TyG-BMI (β: −5.245, 95% CI: −22.555, −1.065, *P*: 0.043) and TyG-WC (β: −31.464, 95% CI: −91.980, −9.052, *P*: 0.038) than women in the first tertile, which was statistically significant. Although, this significant relationship disappeared after adjusting for potential confounders. Despite the level of other cardiometabolic risk factors, including BMI, BFM, WC, FBS, cholesterol, TG, HDL, LDL, SBP, DBP, ABSI, CRI-I and II, TyG, LAP, and AC, before and after adjustments, was lower in the third tertile of DQI compared to the first tertile, this relationship was not significant (*P*-value > 0.05).

**Table 4:** Association of DQI with cardiometabolic risk factors among overweight and obese women (*n* = 197).

	**T1 (*n* = 66)**	**T2 (*****n*** = **65)**		***P*-value**	**T3 (*****n*** = **66)**		***P*-value**	***P*-trend**
		**β**	**95% CI**		**β**	**95% CI**		
BMI (kg/m^**2**^)							
Crude model	Ref	−0.059	(−1.621,1.504)	0.941	0.137	(−1.492, 1.766)	0.869	0.876
Model 1		0.127	(−1.504, 1.758)	0.879	0.543	(−1.303, 2.389)	0.564	0.575
Model 2		0.297	(−1.305, 1.898)	0.716	0.140	(−1.684, 1.964)	0.880	0.854
**BFM**								
Crude model	Ref	0.346	(−2.854, 3.546)	0.832	0.036	(−3.300, 3.372)	0.983	0.973
Model 1		0.760	(−2.582, 4.102)	0.656	1.290	(−2.494, 5.073)	0.504	0.498
Model 2		1.049	(−2.209, 4.306)	0.528	0.510	(−3.201, 4.220)	0.788	0.744
**WHR**								
Crude model	Ref	−0.001	(−0.022, 0.021)	0.956	−0.008	(−0.031, 0.014)	0.476	0.488
Model 1		0.001	(−0.021, 0.023)	0.955	−0.007	(−0.032, 0.018)	0.580	0.605
Model 2		0.000	(−0.022, 0.022)	0.982	−0.014	(−0.039, 0.011)	**0.073**	**0.047**
**WC (cm)**								
Crude model	Ref	0.407	(−3.446, 4.259)	0.836	−0.233	(−4.250, 3.783)	0.909	0.922
Model 1		0.546	(−3.452, 4.544)	0.789	0.873	(−3.653, 5.399)	0.705	0.700
Model 2		0.585	(−3.381, 4.550)	0.773	−0.424	(−4.940, 4.093)	0.854	0.890
**FBS (mmol/L)**								
Crude model	Ref	−2.532	(−6.753, 1.689)	0.240	−2.673	(−6.968, 1.623)	0.223	0.206
Model 1		−2.536	(−7.001, 1.929)	0.266	−1.793	(−6.674, 3.088)	0.471	0.421
Model 2		−3.024	(−7.688, −1.641)	0.034	−1.358	(−6.517, 3.802)	0.606	0.508
**Cholesterol (mmol/L)**								
Crude model	Ref	−3.721	(−17.970,0.528)	0.609	0.852	(−13.647, 15.350)	0.908	0.945
Model 1		4.974	(−9.151, 19.098)	0.490	6.303	(−9.138, 21.743)	0.424	0.405
Model 2		1.375	(−12.885, 15.635)	0.850	2.802	(−12.972, 18.576)	0.728	0.726
**TG (mmol/L)**								
Crude model	Ref	−10.899	(−38.885, 17.086)	0.445	0.792	(−27.950, 29.533)	0.957	0.986
Model 1		−1.810	(−30.306, 26.687)	0.901	−1.063	(−32.386, 30.261)	0.947	0.938
Model 2		−2.012	(−31.141, 27.117)	0.892	−6.222	(−38.58,9 26.144)	0.706	0.712
**HDL (mmol/L)**								
Crude model	Ref	1.294	(−2.967, 5.555)	0.552	−0.326	(−4.662, 4.010)	0.883	0.925
Model 1		2.453	(−1.995, 6.901)	0.180	2.429	(−2.433, 7.292)	0.328	0.196
Model 2		3.306	(0.004, 7.707)	0.161	3.513	(−0.354, 8.381)	0.157	0.147
**LDL (mmol/L)**								
Crude model	Ref	−4.381	(−14.187, 5.425)	0.381	−2.452	(−12.431, 7.526)	0.630	0.595
Model 1		1.039	(−8.633, 10.711)	0.833	2.247	(−8.326, 12.820)	0.677	0.677
Model 2		0.809	(−8.462, 10.080)	0.864	−1.485	(−11.739, 1.770)	0.077	0.812
**SBP**								
Crude model	Ref	−0.387	(−6.131, 5.357)	0.895	0.902	(−5.089, 6.892)	0.768	0.783
Model 1		0.036	(−6.050, 6.123)	0.991	0.237	(−6.664, 7.137)	0.946	0.948
Model 2		0.941	(−5.321, 7.208)	0.768	−0.080	(−7.208, 7.048)	0.982	0.989
**DBP**								
Crude model	Ref	−2.138	(−6.123, 1.847)	0.293	−1.103	(−5.259, 3.053)	0.603	0.567
Model 1		−1.277	(−5.453, 2.898)	0.549	−0.983	(−5.717, 3.751)	0.684	0.655
Model 2		−0.634	(−5.009, 3.742)	0.777	−1.036	(−6.016, 3.944)	0.684	0.677
**ABSI**								
Crude model	Ref	−3.237	(−0.001, 0.001)	0.960	−0.001	(−0.002, 0.001)	0.370	0.383
Model 1		−8.239	(−0.001, 0.001)	0.901	−0.001	(−0.002, 0.001)	0.487	0.503
Model 2		0.000	(−0.002, 0.001)	0.731	−0.001	(−0.002, 0.001)	0.325	0.335
**AIP**								
Crude model	Ref	−0.063	(−0.170, 0.000)	0.050	−0.005	(−0.115, 0.105)	0.933	0.855
Model 1		−0.045	(−0.156, 0.065)	0.423	−0.051	(−0.172, 0.071)	0.414	0.388
Model 2		−0.049	(−0.158, 0.060)	0.378	−0.086	(−0.207, −0.006)	0.126	**0.027**
**CRI_I**								
Crude model	Ref	−0.169	(−0.583, 0.244)	0.422	0.113	(−0.308, 0.533)	0.599	0.661
Model 1		−0.063	(−0.496, 0.370)	0.175	0.026	(−0.447, 0.499)	0.714	0.945
Model 2		−0.197	(−0.609, 0.015)	0.119	0.122	(−0.578, 0.334)	0.600	0.533
**CRI_II**								
Crude model	Ref	−0.150	(−0.432, 0.131)	0.296	−0.007	(−0.293, 0.280)	0.964	0.900
Model 1		−0.073	(−0.363, 0.217)	0.120	−0.008	(−0.325, 0.309)	0.961	0.918
Model 2		−0.104	(−0.378, 0.101)	0.059	−0.123	(−0.427, 0.180)	0.426	0.397
**CHOLIndex**								
Crude model	Ref	−5.675	(−15.784, 0.034)	0.091	−2.127	(−12.412, 8.159)	0.685	0.634
Model 1		−1.414	(−11.637, 0.809)	0.066	−0.182	(−11.358, 10.994)	0.975	0.951
Model 2		−2.497	(−12.080, 7.085)	0.610	−4.998	(−15.597, 0.000)	0.055	**0.031**
**TyG**								
Crude model	Ref	−0.140	(−0.367, 0.007)	0.066	−0.048	(−0.281, 0.185)	0.684	0.620
Model 1		−0.079	(−0.307, 0.048)	0.074	−0.086	(−0.336, 0.164)	0.501	0.475
Model 2		−0.077	(−0.306, 0.152)	0.510	−0.144	(−0.398, 0.110)	0.077	0.260
**TyG–BMI**								
Crude model	Ref	−5.245	(−22.555, −1.065)	**0.043**	−1.668	(−19.441, 16.104)	0.854	0.821
Model 1		1.739	(−15.546, 19.024)	0.844	2.032	(−16.963, 21.026)	0.834	0.825
Model 2		3.617	(−13.388, 20.622)	0.677	−4.186	(−23.128, 1.757)	0.085	0.736
**TyG–WC**								
Crude model	Ref	−31.464	(−91.980, −9.052)	**0.038**	−13.835	(−68.512, 40.842)	0.620	0.564
Model 1		−10.284	(−71.397, 0.030)	0.052	−1.135	(−62.779, 60.510)	0.971	0.136
Model 2		−25.579	(−80.893, 29.735)	0.365	−54.768	(−110.920, 1.384)	0.056	0.054
**LAP**								
Crude model	Ref	−8.149	(−25.671, 9.374)	0.362	4.717	(−12.962, 22.396)	0.601	0.669
Model 1		0.915	(−15.986, 17.816)	0.916	8.107	(−10.333, 26.547)	0.389	0.413
Model 2		1.724	(−15.822, 19.269)	0.847	2.747	(−16.554, 22.048)	0.780	0.773
**AC**								
Crude model	Ref	−0.169	(−0.583, 0.244)	0.422	0.113	(−0.308, 0.533)	0.599	0.66
Model 1		−0.063	(−0.496, 0.370)	0.775	0.026	(−0.447, 0.499)	0.914	0.945
Model 2		−0.197	(−0.609, 0.215)	0.349	−0.122	(−0.578, 0.334)	0.600	0.533

ABSI, a body shape index; AC, atherogenic coefficient; AIP, atherogenic index of plasma; BFM, body fat mass; BMI, body mass index; CI, confidence interval; CRI-I, Castelli risk index 1; FBS, fasting blood sugar; HDL, high-density lipoprotein; LAP, lipid accumulation product; LDL; low-density lipoprotein; TG, triglyceride; TyG, Triglyceride-glucose index; WC, waist circumference; WHR, waist-hip ratio.

Binary logistic regression was used.

Model 1: adjusted for age, physical activity, energy intake, and BMI. Note: BMI as a colinear variable was not adjusted for BMI, BFM, WHR, WC, TyG-BMI, Tyg-WC, LAP, and ABSI.

Model 2: adjusted for model 1 further with education, socioeconomic status, and supplement use.

Bold values indicate *P*-value < 0.05 significant.

## Discussion

This cross-sectional study was conducted to evaluate the association between DQI and cardiometabolic risk factors and ABSI in Iranian women. Accordingly, our findings showed that a high-quality diet in women, assessed by adherence to the DQI, was inversely associated with cardiometabolic risk factors, including WHR, AIP, CHOLINDEX, TyG-BMI, and TyG-WC. There were no significant associations between DQI and other cardiometabolic risk factors and anthropometric indices, including ABSI in women.

Previous studies have shown that a high-quality diet can contribute to the maintenance of cardiovascular health in young and middle-aged adults (42–44). For instance, following a modified Mediterranean diet in the Coronary Artery Risk Development in Young Adults (CARDIA) study led to a reduction of metabolic disorders in middle-aged participants (43). Moreover, higher Alternate Healthy Eating Index scores in the China Health and Nutrition Survey (CHNS) study were related to lower odds of disorders, such as diabetes, and low LDL levels in male participants (45). In a cross-sectional study, it was demonstrated that a higher DASH score was associated with lower insulin resistance and lower serum levels of LDL, HDL, and VLDL, while subjects in the highest DASH quartile had lower odds of metabolic syndrome (46). In the cross-sectional Australian Health Survey, higher scores on the Dietary Guideline Index (DGI) were associated with lower glucose, body mass index, and waist circumference (47). On the other hand, a systematic review and meta-analysis demonstrated that there was no considerable relation between dietary diversity score and most CMRs (48). However, little is known regarding the association between DQI and cardiometabolic risk factors and anthropometric indices.

In a cohort study, a 13-year follow-up among 4,390 adult participants indicated that lower scores of DQI were associated with higher scores of impaired HDL, FBG, LDL, and WC (49). In a cross-sectional study, which was carried out in 2021, it was observed that DQI score was inversely associated with serum level of TC, but positively associated with HDL (50). Alkerwi et al., in the Observation of Cardiovascular Risk Factors in Luxembourg (ORISCAV-LUX) study, found an inverse association between DQI score and total cholesterol, LDL, and HDL levels (51). In a cohort study among 451 participants, after 6.7 years of follow-up, a positive association was found between DQI score and HDL, although only in male participants (52). In a study by Kim et al., DQI had a significant association with fasting plasma glucose in diabetic patients (53). Moreover, results of a 10-year follow-up cohort study, across 10 European countries with over 450,000 participants, indicated that DQI scores were inversely associated with cardiovascular disease mortality and its risk factors, including blood lipids and glucose (54).

Based on our results, the DQI score was not related to the serum glycemic and lipid profile markers; however, the DQI score was inversely associated with CHOLINDEX, TyG-BMI, and TyG-WC, which are related indices to insulin resistance and lipid parameters. In a cross-sectional study conducted on patients with type 2 diabetes, there was no significant relationship between DQI scores and glycemic status and lipid profile parameters in diabetic patients (55). In addition, Daneshzad et al. did not observe any significant association between DQI and CVD risk factors in a cross-sectional study on diabetic women (56). However, in the Korea National Health and Nutritional Examination Survey, higher scores of DQI in middle-aged and older women were associated with a lower risk of body composition abnormalities (57), while Gregory et al.'s DQI was positively associated with BMI and WC (58). Nevertheless, in a cross-sectional study from the results of the Tehran Lipid and Glucose Study, no significant association was observed between DQI and BMI and WC (59). In this study, our results suggested that DQI was inversely associated with WHR, but there was no association between DQI and BMI, BFM, and WC. According to the extant literature, possible reasons for the inconsistency of the published results could be related to the differences in the sample size, different designs of the studies, and differences in the health status of participants.

The present results of the study showed that women with the highest score of DQI had a higher intake of whole grains, fruits, vegetables, legumes, and total fiber, in addition to a lower intake of total fat and saturated fatty acids. It has been reported that a high-quality diet is related to a decreased risk of mortality caused by cardiovascular diseases (60). Dietary combinations can have considerable efficacy on insulin sensitivity (61). The types of fat consumption have been investigated and discussed as a factor in cardiovascular (62), as part of nutritional planning, and for the increase of insulin sensitivity (63). The quality of dietary fats can affect the composition of cell membranes and their functions and also cause to alter in insulin signaling pathways in tissues (64). For example, saturated fatty acids can induce insulin resistance by reducing the release of adiponectin and disrupting insulin signaling pathways associated with glucose uptake in adipose tissue. In addition, fatty acids lead to insulin resistance by causing chronic and mild inflammation through inflammatory cytokines, lipids and their metabolites, and reactive oxygen species (65–67). The saturated fatty acid could cause activates serine kinase proteins, leading to inflammatory pathways, and through reduction tyrosine phosphorylation of insulin receptor substrate 1, adverse affect insulin transportation (57). In addition, other negative results of its consumption include formation of antagonists against insulin activity, such as sphingolipids (68), and the expression of nuclear factor kappa B (NF-kB) and cyclooxygenase-2 (COX-2) (69). Intake of complex carbohydrates, such as fiber, and sources rich in polyphenols and antioxidants, including vegetables, fruits, and legumes, can contribute to an ameliorated cardiovascular status, due to their anti-inflammatory properties (70–72). Dietary fiber intake can, through its fermentation by intestinal bacteria and the production of short-chain fatty acids, lead to a reduction in the synthesis of cholesterol in the body (73, 74). The short-chain fatty acids lead to a decrease in the levels of C-reactive protein and interleukin-6 (75, 76). The benefits of polyphenols are related to their anti-inflammatory and antioxidant properties (77) because they can reduce the release of NO and PGE2 by suppressing the expression of iNOS and PGE2, and also reduce the release of IL-1b and TNF-a, an effect that has been related to suppressing activation of the NF-kB pathway (78, 79). Consumption of these products leads to ameliorate regulation of fat and carbohydrate metabolism, improvement of hyperglycemia, a decrease of dyslipidemia, increased sensitivity to insulin, amendment of adipose tissue metabolism, and decrease in oxidative stress (80). One of the most well-known effects of polyphenols on carbohydrate metabolism is the suppression of the function of the key enzymes (including alpha-glucosidase and alpha-amylase) responsible for its digestion in the gastrointestinal tract (81, 82). Some types of polyphenols can interfere with the absorption of glucose from the small intestine by inhibiting sodium-dependent glucose transporters, such as SGLT1 (83, 84). Polyphenolic ingredients regulated at blood sugar level after meals and improved glucose intolerance by facilitating insulin response and reducing the secretion of hormones such as glucose-dependent insulinotropic polypeptide (GIP) and glucagon-like polypeptide-1 (GLP-1) (85, 86). Some polyphenols can regulate the lipolysis mechanism through the induction of adipose tissue lipase, hormone sensitive lipase, enhances gene expression of mitochondrial uncoupling protein 2 (UCP-2) and carnitin palmitoyl transferase-1 (CPT-1) (87, 88). Most polyphenolic compounds, because of their particular structure, have been known as antioxidant phytochemicals (89). They improve the oxidant-antioxidant equilibrium through the endogenous antioxidant system. These bioactive factors diminish lipid peroxidation and increase the total antioxidant capacity of plasma. In addition, they induce enzymes including superoxide dismutase, catalase, and glutathione peroxidase (90).

This study represents a novel addition to the literature, where we elucidate the relationship between DQI and cardiometabolic risk factors in obese and overweight women. Indeed, among the strengths of our study, unlike most of the studies conducted on the relationship between DQI and cardiometabolic risk factors, which only used BMI, WC, and usual glycemic and lipid serum parameters, our study also investigated the relationship of other atherogenic indices, lipid ratios, and anthropometric indices. Nevertheless, despite the strength and novelty of this study, some limitations should be noted. First, the sample included only women; although this was a purposeful decision, based on the dearth of data in the literature. In addition, since this study was performed only in one province, the results cannot be attributed to the total population. Furthermore, using FFQ makes measurement errors inevitable; these largely emanant from recall and other subjective biases. Finally, because of the cross-sectional design of the study, causal inferences cannot be made.

## Conclusion

In this study, we showed that the higher DQI score was associated with lower WHR, AIP, CHOLINDEX, TyG-BMI, and TyG-WC in overweight and obese women. However, more studies are needed to confirm these findings and elucidate their mechanistic etiology in the future.

## Data availability statement

The raw data supporting the conclusions of this article will be made available by the authors, without undue reservation.

## Ethics statement

Ethics approval and consent to participate was presented in article. Each participant was informed completely regarding the study protocol and provided a written and informed consent form before taking part in the study. The study protocol was approved by the ethics committee of Tehran University of Medical Sciences (TUMS) with the following identification (R.TUMS.VCR.REC.1395.1234.).

## Author contributions

AMo, AMi, and KM designed the search. AMi and MG conducted the sampling. FS performed statistical analysis. AMo, AA, and CC wrote the manuscript. CC and YA revised the article. KM and AMi have responsibility for the final content. All authors have read and approved the final manuscript.
